# Lamin AC Cardiomyopathy, the Next Masquerader: Laminopathy Mimicking Cardiac Sarcoidosis

**DOI:** 10.7759/cureus.80563

**Published:** 2025-03-14

**Authors:** Hanad Bashir, Cassady Palmer, Abhimanyu Reddy, Wojciech Mazur, Thomas OBrien

**Affiliations:** 1 Cardiovascular Medicine, The Christ Hospital, Cincinnati, USA; 2 Internal Medicine, The Christ Hospital, Cincinnati, USA

**Keywords:** cardiac magnetic resonance, cardiac sarcoidosis, fluorodeoxyglucose positron emission tomography, lamin ac dilated cardiomyopathy, late gadolinium enhancement

## Abstract

Phenotypic expression of lamin AC-dilated cardiomyopathy (LMNA-DCM) and cardiac sarcoidosis (CS) at times presents with similar clinical findings, including arrhythmia, conduction abnormalities, and heart failure. Lamin AC-dilated cardiomyopathy is one of the most common causes of genetic DCM with an autosomal dominant inheritance pattern. Contrastingly, a smaller proportion of sarcoid cases present with isolated cardiac involvement, highlighting the intricate nature of this systemic disease. This paper presents three cases highlighting potential pitfalls in the clinical diagnosis and management of laminopathies. Also highlighted is the need for extreme caution when endomyocardial biopsies are equivocal and there is sole reliance on cardiac imaging to establish diagnostic criteria. In addition, this case series highlights the imperative role of genetic testing during the initial workup for establishing the accurate etiology in the setting of cardiomyopathy. The strikingly similar clinical characteristics of LMNA-DCM and CS underscore the necessity for a robust diagnostic approach, including early integration of genetic testing, as this approach holds great promise for improving outcomes and the quality of life for those grappling with these challenging cardiac conditions. Innovative strategies, including comprehensive advanced imaging protocols and precision medicine, promise to advance our comprehension and management of both disease entities.

## Introduction

Sarcoidosis is a multisystem inflammatory disease characterized by non-caseating granulomas in various organs, most commonly the lungs, lymph nodes, skin, and eyes. Its cause is unknown but is thought to result from an unidentified antigenic trigger. Symptoms vary based on organ involvement, with pulmonary manifestations like dry cough and dyspnea being the most common. Extrapulmonary symptoms include skin lesions, uveitis, fatigue, joint pain, and, in severe cases, cardiac or neurological complications. Diagnosis requires clinical correlation, imaging, and biopsy to exclude other granulomatous diseases [1]. Although the diagnostic criteria for cardiac sarcoidosis (CS) are based on the Heart Rhythm Society (HRS) expert consensus statement, which considers both histological and clinical pathways, there is no consensus on whether all diagnostic criteria must be fully met for a definitive diagnosis [2]. The HRS criteria allow for a “probable” diagnosis of CS if extracardiac tissue findings are compatible with sarcoidosis, combined with at least one major clinical or imaging marker. However, this criterion is limited by its reliance on tissue biopsy to confirm the presence of non-caseating granulomas; without this confirmation, other causes of arrhythmogenic cardiomyopathy cannot be excluded. In 2016, the Japanese Circulation Society (JCS) guidelines outlined that sarcoidosis can be diagnosed in the absence of histological confirmation if two organ systems exhibit clinical findings consistent with sarcoidosis and at least two of five classic diagnostic tests yield positive results [3]. These diagnostic markers include bilateral hilar lymphadenopathy, elevated serum angiotensin-converting enzyme activity or serum lysozyme levels, increased serum interleukin-2 receptor levels, significant tracer accumulation in 67Ga citrate scintigraphy or 18-fluoro-deoxyglucose positron emission tomography (18F-FDG) PET scans, or a bronchoalveolar lavage fluid CD4/CD8 ratio greater than 3.5. However, the JCS guidelines maintain that a histological diagnosis is required for cases of isolated CS [3].

The prevalence of cardiac involvement in patients with pulmonary or ocular sarcoidosis has not been fully established, with some studies in the United States estimating rates as high as 20% to 29%. However, prevalence estimates may vary across countries, particularly due to detection challenges in underserved regions where access to advanced imaging and specialized care is limited. Additionally, disparities in cardiovascular care for patients with systemic sarcoidosis, driven by social determinants such as healthcare accessibility, socioeconomic status, and racial or geographic inequities, may further influence reported prevalence and outcomes [4,5]. A correct diagnosis can be further complicated by the low diagnostic yield of endomyocardial biopsy due to sampling error, often leading to a missed diagnosis. Patients suspected of having CS frequently undergo multiple imaging modalities, including transthoracic echocardiography, cardiac magnetic resonance (CMR) imaging, and FDG-PET, yet findings can be nonspecific or easily overlooked. Approximately 40% of all cases of dilated cardiomyopathy (DCM) are associated with an underlying genetic variant, with autosomal dominant lamin AC (LMNA)-related DCM accounting for 5% to 10% of these cases [6]. Lamin AC-related DCM is the second most common cause of inherited DCM and results from defects in the lamin A and lamin C intermediate proteins of the nuclear lamina. Clinically, this cardiomyopathy commonly presents with conduction abnormalities, including heart block, sinus node dysfunction, arrhythmias, and heart failure. A definitive diagnosis requires genetic testing. Lamin AC-related DCM demonstrates age-related penetrance, typically manifesting in the third and fourth decades of life, with penetrance exceeding 90% to 95% by the seventh decade [7].

## Case presentation

Case series

Patient 1

**Figure 1:**
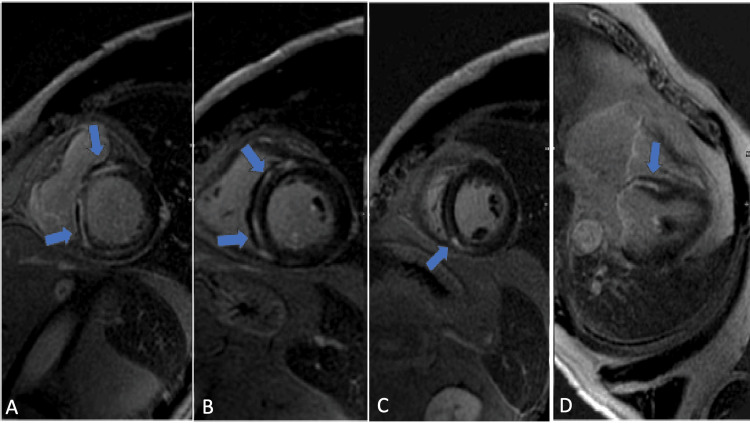
Cardiovascular MRI demonstrates mid-myocardial delayed enhancement (gadolinium) involving the basal anterior (A), anteroseptal, inferoseptal (B), inferior, and inferoseptal segments (C), as well as the mid-anteroseptal segment (D) (blue arrows).

Patient 1, a 51-year-old man with a family history of sudden cardiac death, presented with palpitations and lightheadedness. His vital signs were notable for a mildly elevated heart rate and normal blood pressure. An electrocardiogram demonstrated sinus tachycardia with a heart rate of 105 bpm. He had a history of paroxysmal atrial fibrillation and had previously undergone a Cox-Maze procedure. Although asymptomatic for six years, he began experiencing sustained ventricular tachycardia. A comprehensive clinical workup, including left and right coronary angiography, was performed to assess for coronary artery disease, which yielded normal results. Cardiac magnetic resonance imaging was ordered for tissue characterization and revealed multifocal myocardial inflammation, with active inflammation observed in the basal to mid-inferior, inferoseptal, inferolateral, and mid-wall segments (Figure 1).

**Figure 2:**
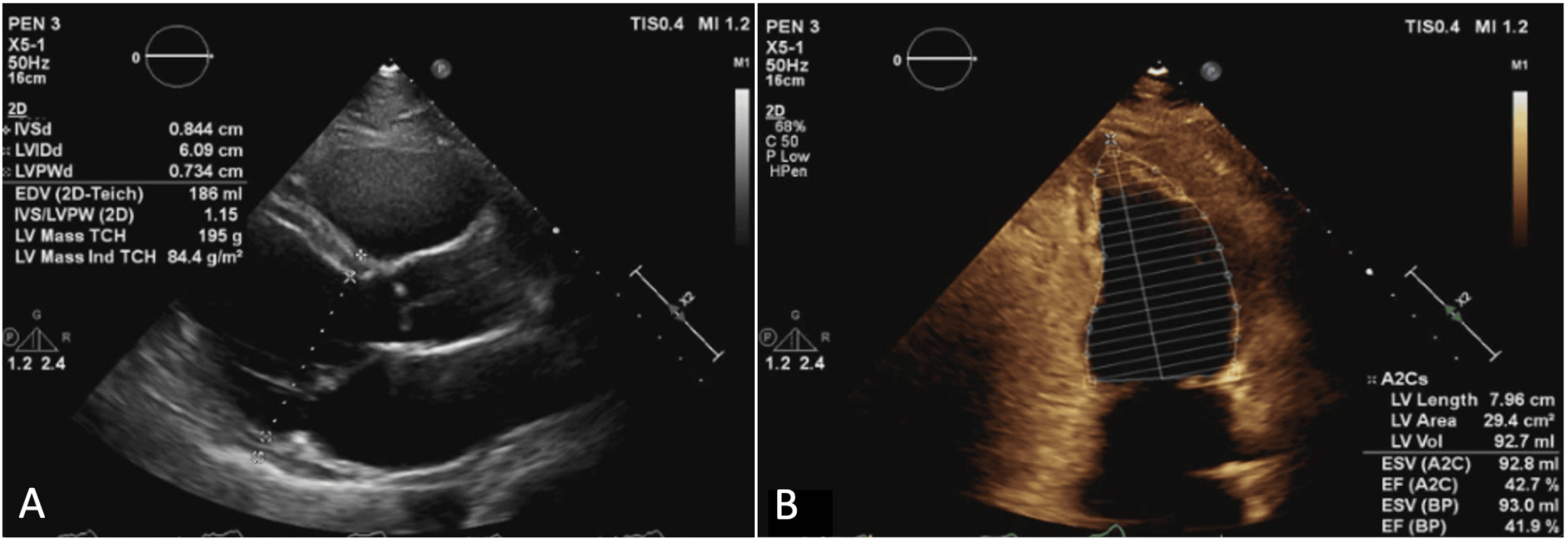
Parasternal long-axis (A) and two-chamber view (B) TTE demonstrate a dilated left ventricular cavity with an end-diastolic diameter measuring 6.1 cm; however, relative wall thickness and mass index are normal. The LVEF is 42% by Simpson’s biplane method. TTE: transthoracic echocardiography; LVEF: left ventricular ejection fraction

T2-weighted images displayed an elevated myocardial-to-skeletal muscle ratio, suggesting myocardial edema. Transthoracic echocardiography (TTE) determined a left ventricular ejection fraction (LVEF) of 42% using Simpson’s Biplane method (Figure 2).

**Figure 3:**
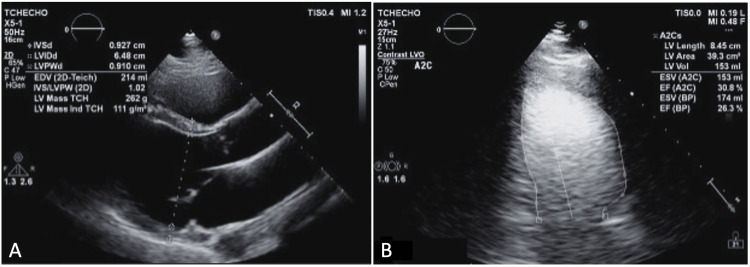
Parasternal long-axis (A) and two-chamber view (B) TTE demonstrates a dilated LV cavity with end-diastolic diameter measuring 6.5 cm; however, relative wall thickness and mass index are normal. LVEF of 26% using the Simpson biplane method. TTE: transthoracic echocardiography; LV: left ventricular; LVEF: left ventricular ejection fraction

Mild diffuse hypokinesis with regional variations was noted. There was no evidence of valvular regurgitation or stenosis. The FDG-PET imaging performed at an external facility demonstrated abnormal perfusion in the anteroseptal and apical inferior walls, along with extensive FDG uptake. Given the strong suspicion of an ongoing inflammatory process, prednisone and methotrexate were initiated. Endomyocardial biopsies were obtained to investigate the cause of inflammation. Despite multiple samples, none confirmed sarcoidosis. However, a diagnosis of probable CS was established based on the patient meeting at least two major criteria set by the Japanese Ministry of Health and Welfare (JMHW), which includes fatal ventricular arrhythmia (VT), abnormal FDG-PET uptake, and LVEF <50%. A subsequent TTE revealed further LVEF decline to 26%, with significant right ventricular dysfunction (Figure 3).

Consequently, a biventricular implantable cardioverter defibrillator (ICD) was implanted. At a two-year follow-up, the patient experienced recurrent ventricular tachycardia (VT). A repeat FDG-PET scan at an external facility showed no FDG uptake or active inflammation. Following these findings, genetic testing was performed, revealing a pathogenic LMNA gene variant (c.1436del (p. Leu479Argfs*11)). During genetic counseling, the patient disclosed a maternal family history of sudden cardiac death, though no confirmed genetic cardiomyopathy diagnoses were reported. Immunomodulatory therapy was gradually tapered, and the patient was placed on the heart transplant waitlist.

Patient 2

**Figure 4:**
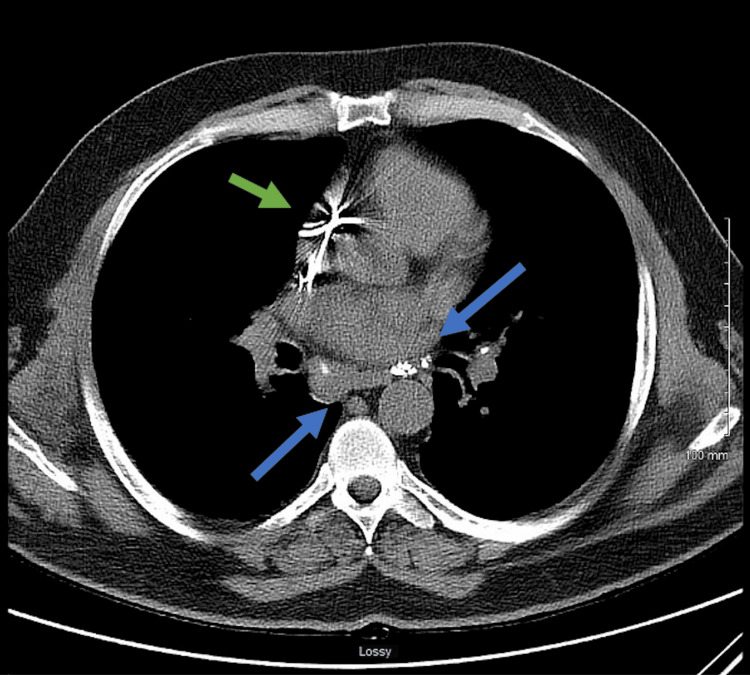
Non-contrast CT chest demonstrates lymphadenopathy, characterized by focal calcifications prominently within the subcarinal space and on both sides of the hilar regions, as indicated by the blue arrows. Defibrillator leads, with wires, are clearly visible within the right atrium and ventricle (green arrow).

Patient 2, a 53-year-old man with non-ischemic DCM, chronic heart failure with reduced ejection fraction, coronary artery disease, and pulmonary sarcoidosis confirmed by cardiac CT presented for a follow-up evaluation. A resting perfusion scan from 2012 revealed decreased perfusion in the apex and apical segment of the lateral wall. A non-gated resting myocardial viability FDG-PET scan showed increased uptake in the anterior wall, anterior apical wall, inferior wall, and septum, with the most significant involvement in the septum and lateral wall (Figure 4).

The FDG-PET imaging demonstrated diffuse myocardial FDG uptake, a moderately dilated left ventricle, and lymphadenopathy with focal calcifications, particularly within the subcarinal space and hilar regions bilaterally (Figure 4).

**Figure 5:**
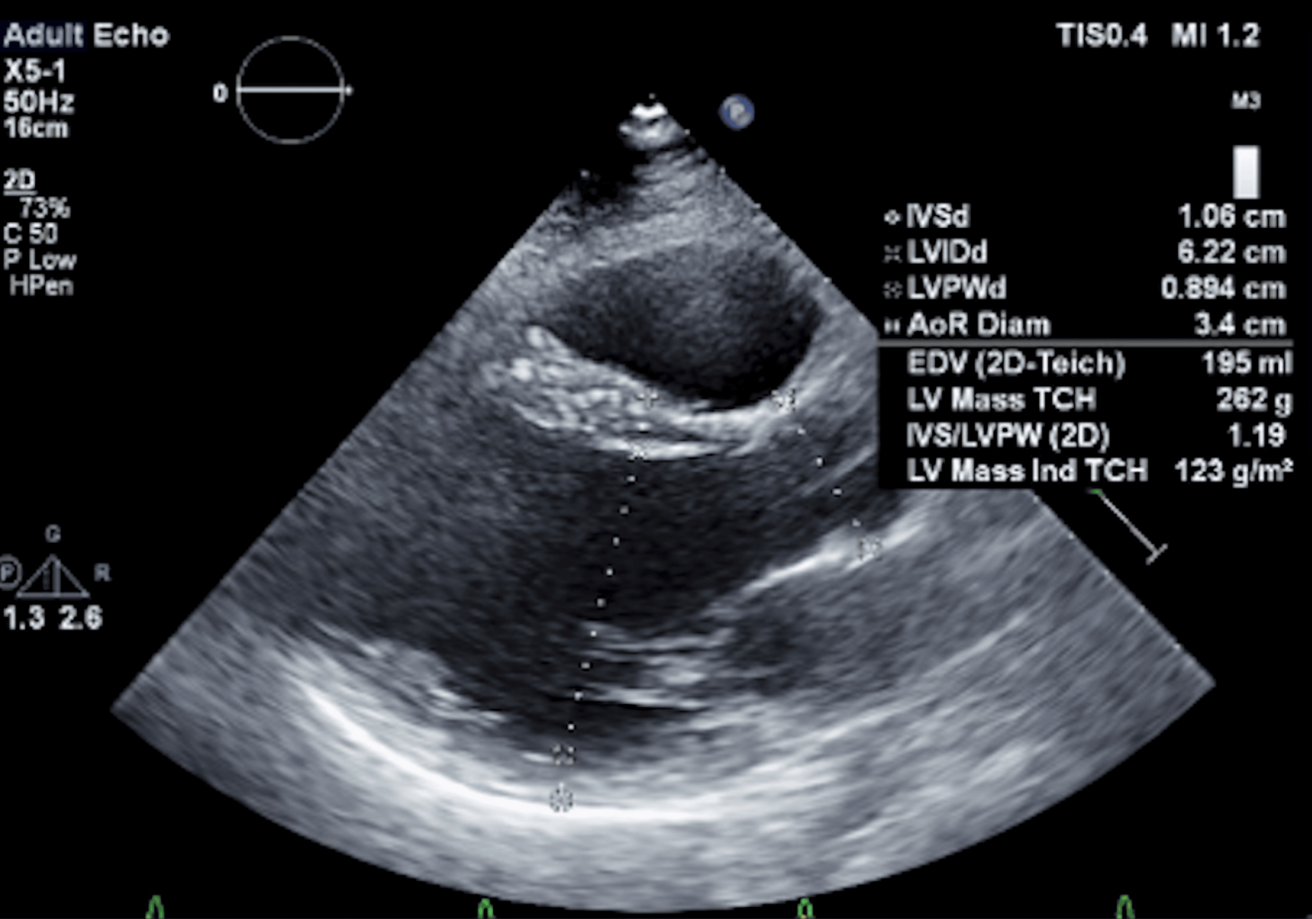
Parasternal long-axis TTE demonstrated eccentric hypertrophy (increased left ventricular mass index and normal relative wall thickness). Dynamic heart 3D modeling of the left ventricle revealed an ejection fraction of 38%. TTE: transthoracic echocardiography

A TTE revealed eccentric hypertrophy (increased left ventricular mass index with normal relative wall thickness), and a 3D LVEF of 38% was calculated (Figure 5).

**Figure 6:**
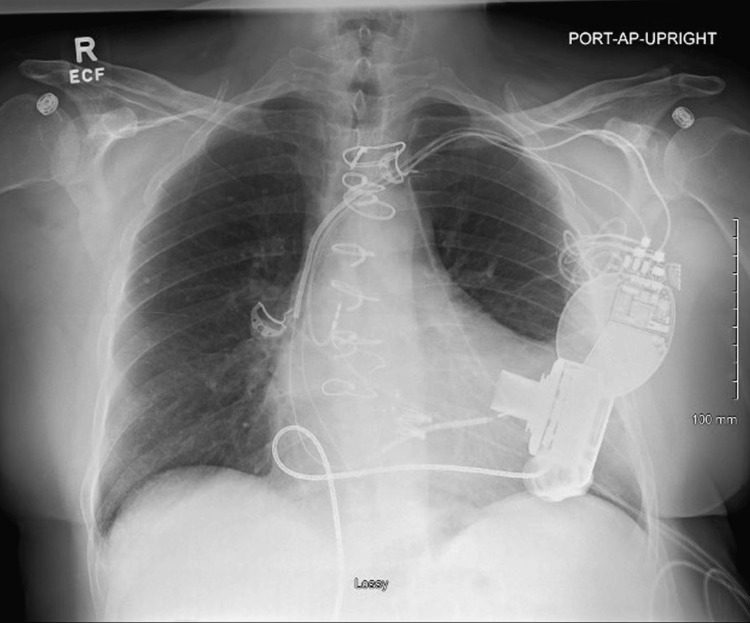
The portable AP X-ray view shows the left ventricular assist device in a stable position. Additionally, the left subclavian pacemaker and ICD are both confirmed to be in stable positions. AP: anteroposterior; ICD: implantable cardioverter defibrillator

Due to high clinical suspicion of CS despite the absence of a definitive endomyocardial biopsy, the patient was initiated on prednisone, which was maintained for several years before transitioning to azathioprine therapy. During follow-up, the patient experienced recurrent VT episodes, which persisted despite undergoing ablation. A whole-body repeat PET scan demonstrated FDG uptake limited to the lung base. In 2018, a cardiac viability PET scan revealed moderate left ventricular dilation, and FDG uptake in the basal inferolateral segment, apical inferior segment, and apex (Figure 6).

The remaining myocardium showed normal viability with no evidence of scarring. The LVEF was estimated at 30%, with 18% myocardial scarring but no active inflammation. While cardiac arrhythmias ceased, the patient’s heart failure symptoms worsened, ultimately necessitating left ventricular assist device implantation without complications (Figure 6).

Notably, the patient’s son presented separately with exertional syncope and was diagnosed with DCM but without evidence of sarcoidosis. Genetic testing revealed both father and son carried the pathogenic LMNA variant c.1412>A (p. Arg471His). Despite two grandchildren with a cardiac history, no additional familial cases of genetic cardiomyopathy were confirmed through cascade testing.

Patient 3

**Figure 7:**
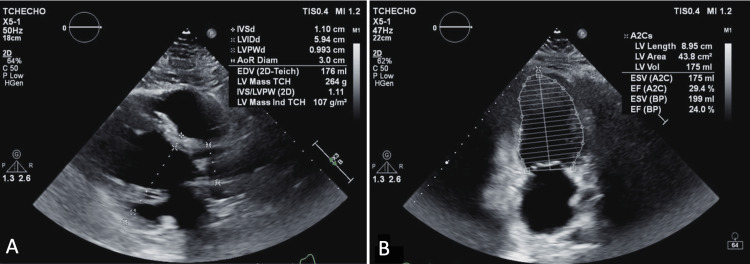
Parasternal long-axis (A) and two-chamber view (B) TTE demonstrated normal LV wall thickness and mass index. The LVEF is calculated at 24% by Simpson’s biplane method. TTE: transthoracic echocardiography; LV: left ventricular; LVEF: left ventricular ejection fraction

Patient 3, a 32-year-old man with a history of hypertension, morbid obesity, obstructive sleep apnea, and tobacco use, presented with decompensated heart failure and newly diagnosed DCM. A TTE demonstrated normal left ventricular wall thickness and mass index, with an LVEF of 24% calculated by Simpson’s Biplane method (Figure 7).

**Figure 8:**
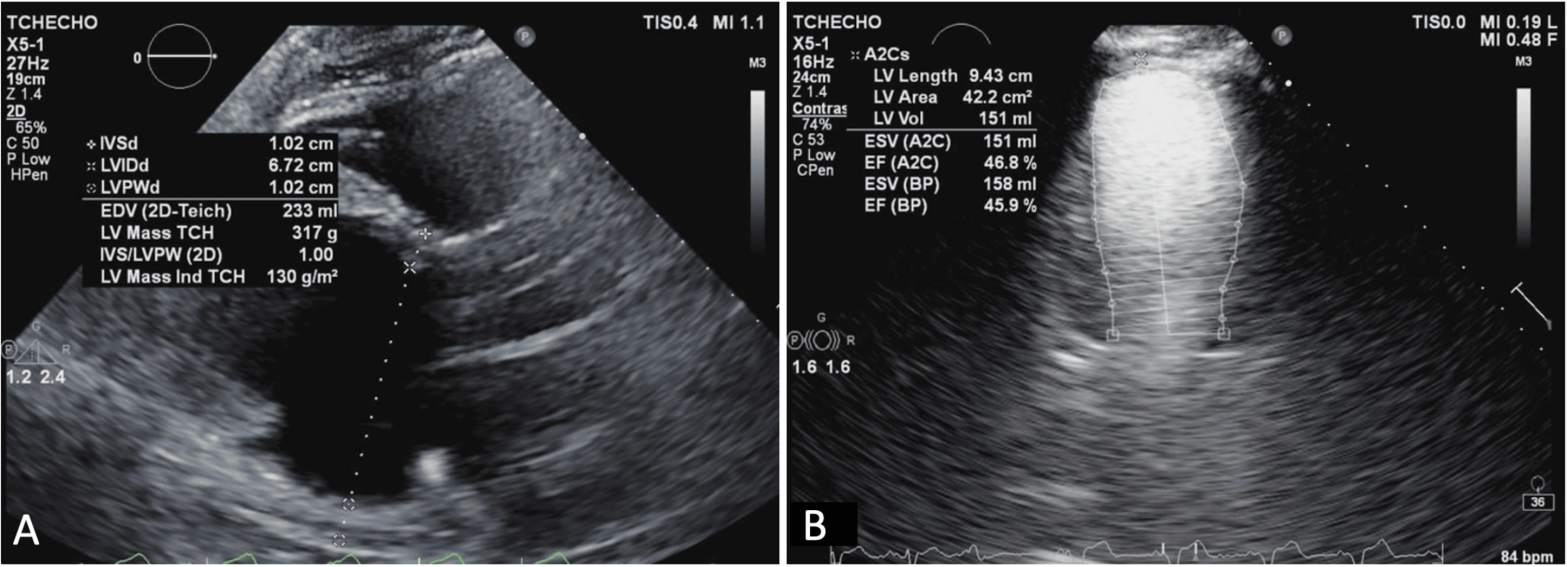
Parasternal long-axis (A) and two-chamber view (B) TTE demonstrate eccentric hypertrophy (normal LV wall thickness and increased mass index). An increased end-diastolic diameter measuring 6.7 cm is noted. Improvement in LVEF at the time of repeat echo is now calculated at 46% by Simpson’s biplane method. TTE: transthoracic echocardiography; LV: left ventricular; LVEF: left ventricular ejection fraction

Guideline-directed medical therapy (GDMT) was initiated, resulting in significant improvement in ejection fraction; however, the patient was lost to follow-up. He was later readmitted with worsening heart failure symptoms and reduced ejection fraction despite continued GDMT. Repeat TTE demonstrated an increased end-diastolic diameter measuring 6.7 cm and eccentric hypertrophy (normal left ventricular wall thickness with an increased mass index). The LVEF improved to 46% per Simpson’s biplane method (Figure 8).

**Figure 9:**
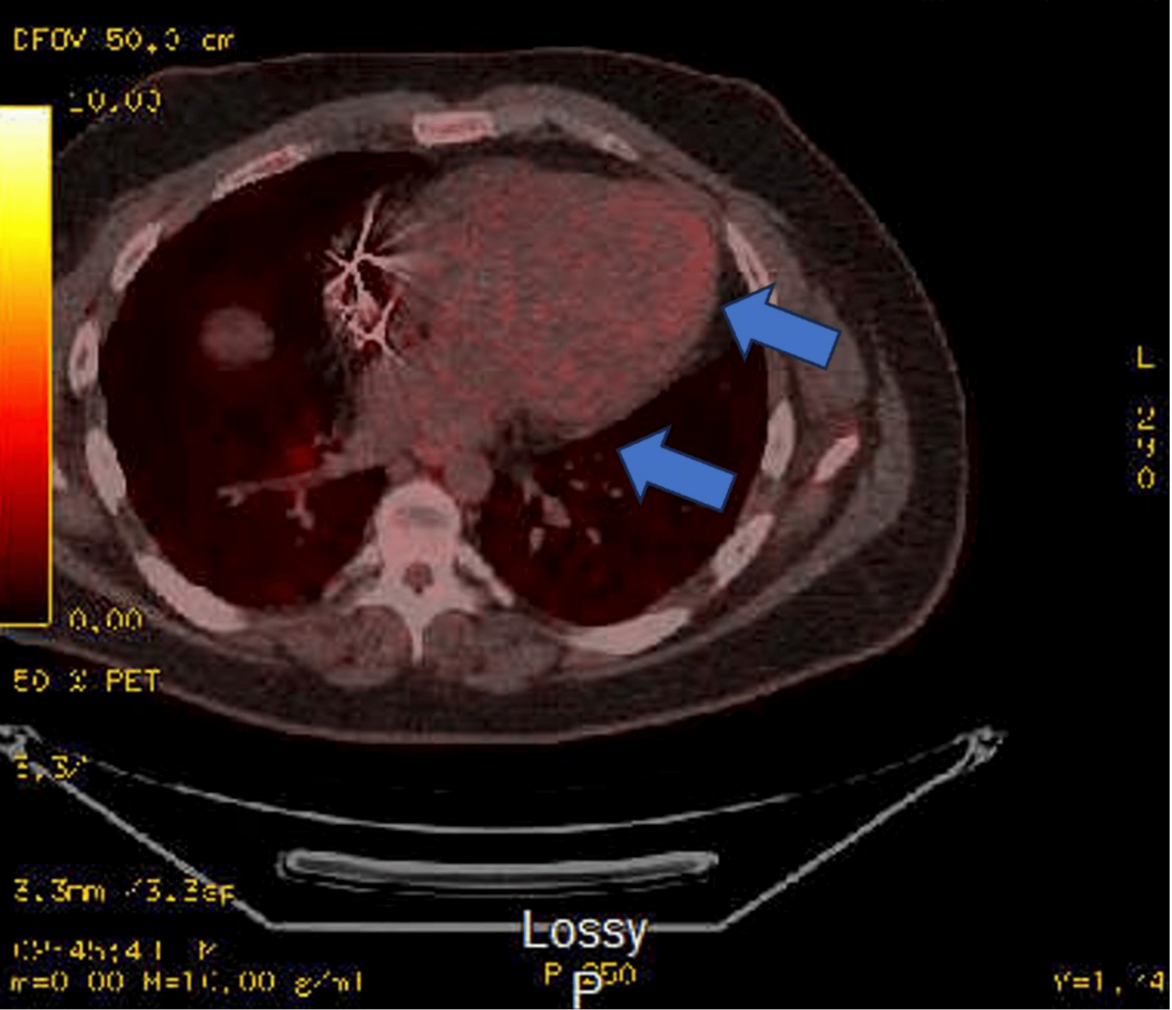
The FDG-PET myocardial metabolic imaging demonstrates focal areas of FDG uptake indicative of metabolic activity at the level of the cardiac apex, apical anterior, apical lateral, and anterolateral walls (blue arrow). A smaller region of FDG uptake is also observed in the inferolateral wall. FGD-PET: fluorodeoxyglucose positron emission tomography

An ECG revealed a new left bundle branch block (LBBB), prompting biventricular ICD implantation. In 2017, an FDG-PET scan showed FDG uptake in the apex, apical anterior, apical lateral, and anterolateral walls, along with a small focus in the inferior wall. Additionally, rubidium resting myocardial perfusion images revealed perfusion defects corresponding to the apex, apical anterior, apical lateral, mid-anterolateral, and apical inferior segments (Figure 9).

**Figure 10:**
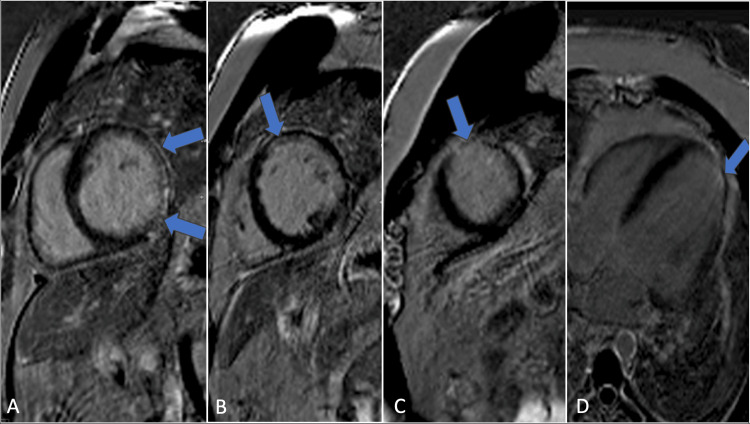
Cardiovascular MRI demonstrated subendocardial delayed enhancement at all levels, including the base (A), mid-cavity (B), anterolateral (C), and apical lateral segments (D). The delayed enhancement was greater than 50% transmurally (blue arrows). Additionally, transmural delayed enhancement was observed in the anterior apical segment (D).

The LVEF was estimated at 20%, with severe diffuse hypokinesis. Cardiac magnetic resonance imaging demonstrated moderate left ventricular dilation, global hypokinesis, and an LVEF of 30%. It also showed multifocal subendocardial delayed enhancement in the basal, mid-cavity, and apical segments, with more than 50% transmural involvement and transmural delayed enhancement in the anterior apical segment (Figure 10).

An MRI revealed incidental lymphadenopathy and pulmonary nodules. The TTE findings showed a markedly dilated left ventricle with eccentric hypertrophy and a mildly increased ventricular mass. The LVEF was measured at 46% by Simpson’s biplane method, with regional wall motion abnormalities in the apical septal, apical lateral, and apical cap regions. A probable diagnosis of CS was established based on JMHW guidelines, meeting at least two major criteria (LVEF <50% and serial FDG-PET scans demonstrating FDG uptake) and minor criteria (LBBB), despite negative biopsies from lymph nodes, pulmonary, and cardiac samples. Immunosuppressive therapy was initiated, and the patient was later maintained on azathioprine. Family history was significant for heart failure in the father, who had undergone heart transplantation. Genetic testing confirmed a pathogenic LMNA variant (c.1412G>A (p.Arg471His)). Given the positive genetic findings, the patient was referred for heart transplantation.

## Discussion

The clinical presentations of CS and LMNA-DCM share overlapping features, including conduction abnormalities and heart failure. Differentiating these two disease entities is challenging due to the absence of randomized controlled trials validating whether all diagnostic criteria are necessary for confirming CS. Additionally, cardiac imaging often yields equivocal findings, contributing to diagnostic uncertainty. A definitive diagnosis of CS requires a positive endomyocardial biopsy; however, the patchy distribution of sarcoid granulomas leads to a low sensitivity of this technique due to intrinsic sampling error. Given that subclinical CS accounts for approximately one-quarter of reported cases, advanced cardiac imaging remains the primary tool for assessing cardiac involvement. Among imaging modalities, CMR and FDG-PET imaging are the two principal techniques used to evaluate inflammatory and infiltrative disease processes [7].

Fluorodeoxyglucose-positron emission tomography, a hybrid imaging technique, is a noninvasive test useful for detecting inflammatory foci in vasculitis. Unlike conventional anatomical imaging, it can identify early inflammation. Cardiac MRI, with its robust tissue characterization capabilities, can detect myocardial fibrosis and assess tissue viability through delayed enhancement techniques [8].

Despite these advantages, the specificity of both modalities remains a challenge, as several conditions, including viral myocarditis, arrhythmogenic right ventricular cardiomyopathy, LMNA mutations, hypertrophic cardiomyopathy, idiopathic granulomatous myocarditis, systemic lupus erythematosus, and other autoimmune or inflammatory conditions, can present with similar imaging findings. Consequently, misdiagnosis can occur when imaging findings alone are interpreted without considering broader clinical and genetic factors [9].

The updated diagnostic approach for CS addresses the limitations of historical criteria by implementing a structured algorithm. The process begins with clinical suspicion, followed by CMR. If CMR is inconclusive or normal but clinical suspicion remains high, FDG-PET is performed. A definitive diagnosis typically requires histologic confirmation from cardiac or extracardiac tissue. In cases where tissue diagnosis is unavailable but isolated CS is suspected based on CMR, PET, and clinical indicators, genetic testing is considered [10].

This case series highlights the risk of misdiagnosing CS when relying solely on noninvasive imaging findings suggestive of active inflammation and scarring. While imaging plays a crucial role in detecting subclinical CS, it is essential for diagnosing all forms of the disease, not just subclinical cases. For example, although Patient 2 had biopsy-confirmed extracardiac sarcoidosis, no endomyocardial biopsy was performed despite PET findings consistent with CS, as indicated by FDG uptake. However, follow-up imaging showed no evidence of active inflammation, which was attributed to disease progression rather than ongoing sarcoidosis. Conversely, imaging findings for Patients 1 and 3 were also suggestive of CS, but subsequent genetic testing led to a diagnosis of LMNA-DCM. These patients had a strong family history, reduced ejection fraction, and conduction abnormalities. Additionally, both tested negative for viral etiologies, including hepatitis and Epstein-Barr virus (EBV), further supporting a genetic rather than inflammatory etiology.

This underscores the limitations of relying solely on imaging for CS diagnosis, as findings such as FDG uptake and myocardial scarring are nonspecific and may overlap with other conditions, including LMNA-DCM. A comprehensive approach incorporating clinical history, genetic testing, and, when feasible, tissue diagnosis is essential to avoid misdiagnosis and guide appropriate management.

## Conclusions

This case series underscores the critical need for incorporating genetic testing early in the diagnostic evaluation of patients suspected of having CS. The overlapping clinical and imaging features of CS and LMNA-DCM make definitive diagnosis challenging, particularly when endomyocardial biopsy results are inconclusive. In this study, genetic testing revealed LMNA mutations in two patients initially suspected of having CS based on imaging findings. Additionally, follow-up imaging in a patient with biopsy-confirmed extracardiac sarcoidosis demonstrated no active cardiac inflammation, suggesting disease progression rather than active CS. The findings highlight the importance of a multimodal diagnostic approach, integrating CMR, FDG-PET, and genetic testing to reduce the risk of misdiagnosis. Early genetic evaluation can prevent unnecessary immunosuppressive therapy and guide appropriate treatment strategies. Moving forward, a structured diagnostic algorithm that incorporates genetic screening alongside conventional imaging and histological assessment may improve diagnostic accuracy and optimize patient management in suspected cases of CS and infiltrative cardiomyopathies.
